# Previously differentiated medial vascular smooth muscle cells contribute to neointima formation following vascular injury

**DOI:** 10.1186/2045-824X-6-21

**Published:** 2014-10-01

**Authors:** Brian Paul Herring, April M Hoggatt, Christopher Burlak, Stefan Offermanns

**Affiliations:** 1 Department of Cellular and Integrative Physiology, Indiana University School of Medicine, Indianapolis, IN 46202, USA; 2 Department of Surgery, Indiana University School of Medicine, Indianapolis, IN 46202, USA; 3 Current address: Schultz Diabetes Institute, Department of Surgery, University of Minnesota, Minneapolis, MN 55455, USA; 4 Department of Pharmacology, Max-Planck-Institute for Heart and Lung Research, Ludwigstr. 43, 61231 Bad Nauheim, Germany

**Keywords:** Vascular smooth muscle, Neointima, Smooth muscle myosin, Smooth muscle α-actin

## Abstract

**Background:**

The origins of neointimal smooth muscle cells that arise following vascular injury remains controversial. Studies have suggested that these cells may arise from previously differentiated medial vascular smooth muscle cells, resident stem cells or blood born progenitors. In the current study we examined the contribution of the previously differentiated vascular smooth muscle cells to the neointima that forms following carotid artery ligation.

**Methods:**

We utilized transgenic mice harboring a cre recombinase-dependent reporter gene (mTmG). These mice express membrane targeted tandem dimer Tomato (mTomato) prior to cre-mediated excision and membrane targeted EGFP (mEGFP) following excision. The mTmG mice were crossed with transgenic mice expressing either smooth muscle myosin heavy chain (*Myh11*) or smooth muscle α-actin (*Acta2*) driven tamoxifen regulated cre recombinase. Following treatment of adult mice with tamoxifen these mice express mEGFP exclusively in differentiated smooth muscle cells. Subsequently vascular injury was induced in the mice by carotid artery ligation and the contribution of mEGFP positive cells to the neointima determined.

**Results:**

Analysis of the cellular composition of the neointima that forms following injury revealed that mEGFP positive cells derived from either *Mhy11* or *Acta*2 tagged medial vascular smooth muscle cells contribute to the majority of neointima formation (79 ± 17% and 81 ± 12%, respectively).

**Conclusion:**

These data demonstrate that the majority of the neointima that forms following carotid ligation is derived from previously differentiated medial vascular smooth muscle cells.

## Background

Vascular smooth muscle cells (VSMCs) are the major contractile components of the vascular system. They are critically important for regulating blood pressure and flow throughout the vascular system. Unlike skeletal and cardiac muscle cells, VSMCs are remarkably plastic and modulate their phenotype in response to extracellular cues during the development and progression of a variety of diseases including atherosclerosis, hypertension, stenosis following injury and restenosis following vascular interventions. Cardiovascular disease is the leading cause of death among the US population, yet despite intense research efforts a number of basic questions regarding the etiology of cardiovascular disease remain elusive. Classically these diseases are described as being associated with dedifferentiated VSMCs that have decreased expression of proteins required for the normal contractile function, increased expression of extracellular matrix proteins and increased cell proliferation [1]. These proliferating dedifferentiated VSMCs are a major component of neointimal lesions and atherosclerotic plaques. Neointimal VSMCs have been proposed to arise from several sources, including blood and bone marrow derived precursor cells, dedifferentiated medial VSMCs, resident progenitor cells and adventitial fibroblasts. However, recent definitive studies showed a relatively minor contribution of blood and bone marrow derived cells to the neointima or atherosclerotic plaque VSMC population [2-5]. The most widely accepted paradigm that neointimal VSMCs arise from the dedifferentiation and migration of medial VSMCs has been recently challenged [6]. This finding has stimulated much controversy in the field [7] and has prompted us to further investigate the origin of these cells. Using a genetic fate mapping approach with tamoxifen regulated smooth muscle-specific cre recombinase and a dual color cre-dependent reporter gene we unequivocally show that the neointimal SMCs that arise following carotid artery ligation are largely derived from the previously differentiated medial VSMCs.

## Methods

### Transgenic mice and carotid ligation

**Figure 1:**
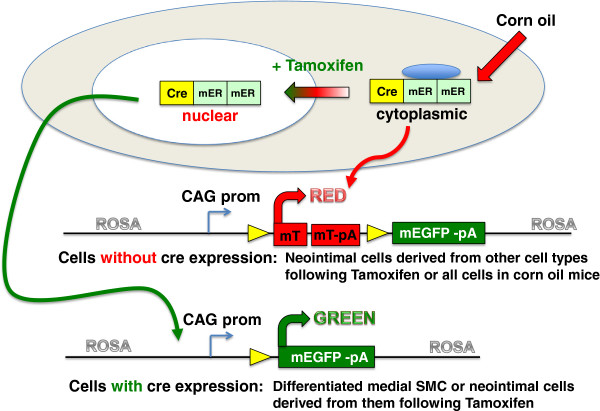
**Schematic representation of the mTmG reporter strain.** The mTmG reporter mice (Jackson strain: B6.129 (Cg)-Gt(ROSA)26Sor^tm4(ACTB-tdTomato,-EGFP)Luo^/J ) contain a single copy of the transgene integrated into the ROSA 26 locus. The transgene cassette is comprised of a chimeric CMV, β-actin promoter driving the expression of a floxed membrane localized Tomato tandem dimer. Following cre-mediated excision the mTomato transgene is removed such that the CAG promoter now drives expression of membrane localized EGFP. The reporter mice were crossed with transgenic mice that express a tamoxifen regulated cre recombinase directed to smooth muscle cells by either the *Myh11* promoter or the *Acta2* promoter. Double heterozygous transgenic mice were used for all experiments.

All animal procedures were performed using procedures approved by the Indiana University School of Medicine Institutional Animal Care and Use committee under protocol number 10310. Smooth muscle myosin heavy chain (*Myh11*) creER(T2)^-/+^ mice [8] (on a C57BL6 background) and smooth muscle α-actin (*Acta2*) creER(T2)^-/+^ mice [9] were crossed with mTmG reporter mice (Jackson strain: B6.129(Cg)-Gt(ROSA)26Sor^tm4(ACTB-tdTomato,-EGFP)Luo^/J) (Figure 1). creER(T2) transgenes were used under agreement from the Institut de Génétique et de Biologie Moléculaire et Cellulaire (IGBMC). Double heterozygous transgenic mice were used for all experiments unless indicated otherwise. At 5-6 weeks of age mice were treated with Tamoxifen (1 mg/mouse IP) or corn oil control, once a day for 5 days. 2 weeks following the last treatment the left carotid artery was ligated as described previously [10]. Prior to surgery mice were anesthetized with intraperitoneal injection of ketamine/xylazine (0.088 mg/gm, 0.012 mg/gm). Following surgery mice were given a single subcutaneous injection of carprofen analgesic (0.5 mg). At various times following ligation mice were sacrificed under anesthesia (intraperitoneal injection of ketamine/xylazine (0.088 mg/gm, 0.012 mg/gm) and the injured and control contralateral carotid arteries were harvested and fixed in 4% paraformaldehyde for 2 hours on ice. Following fixation, vessels were washed in phosphate buffered saline for 3× 5 minutes and then frozen in OCT tissue freezing media (Tissue-Tech) on a bed of dry ice/2-methylbutane. 8 μm cryosections were cut and stored at -80°.

### Histological and immuno-staining

OCT was removed from sections by 3, 5 minute washes in Tris buffered saline (TBS; 100 mM Tris pH7.6, 150 mM NaCl). Slides were mounted in Prolong Gold containing DAPI (Invitrogen) and visualized by confocal microscopy (Olympus Fluoview FV1000). For immuno-fluorescent staining cryosections were permeabilized in 0.2% triton in TBS for 5 minutes, washed in TBS for 5 minutes them blocked in 5% goat serum diluted in TBS at room temperature for 1 hour. Blocked sections were incubated for 4-5 hours at 37° with primary antibodies to, the SM2 isoform of smooth muscle myosin heavy chain (1:500) [11], CD31 (1:100, clone 390, Affymetrix, eBioscience), CD68 (1:200, clone FA-11, AbD Serotec) diluted in 5% goat serum/TBS. Some sections were incubated without primary antibody as a negative control. Following washing in TBS primary antibodies were detected by incubation with anti-rabbit or anti-rat Alexa Fluor 647 (1:10,000, Jackson ImmunoResearch). After washing slides were mounted in Prolong Gold containing DAPI (Invitrogen) and visualized by confocal microscopy.

### Quantitation of mEGFP and mTomato positive cells in lesions

In order to count the number of mEGFP and mTomato positive cells within the neointima of each vessel, a Z-stack series of 1 μm optical sections were obtained from each vessel. mEGFP and mTomato positive cells were counted in each of the optical sections through the entire Z-stack of images, each DAPI positive nuclei was scored as being associated with either an mEGFP or mTomato positive cell. For some sections it was possible to obtain 2 different fields of Z-stack images.

#### Fluorescence activated cell sorting (FACS)

For FACS blood was harvested from mice immediately prior to tissue collection. Blood collected into heparinized tubes was diluted 10 fold into red blood cell lysis buffer (168 mM NH_4_Cl, 10 mM KHCO_3_, 0.1 mM EDTA, pH7.3). Cells were collected by brief centrifugation (5 min, 400×g), washed in the same buffer and recentrifuged. Pelleted cells were resuspended in phosphate buffered saline containing 0.5% bovine serum albumen, 0.5 mM EDTA and subjected to FACS analysis. Red blood cell fragments were excluded based on side scatter analysis and the remaining cells sorted based on their red and green fluorescence. A minimum of 10,000 cells were counted in each sample. For each analysis, cells obtained from a mouse that expresses EGFP ubiquitously (CAG-EGFP: C57BL/6-Tg(CAG-EGFP)131Osb/LeySopJ) were used as a positive control for EGFP-expressing cells. Cells obtained from a cre negative mTmG mouse were used as a positive control for mTomato expressing cells and cells obtained from a nontransgenic mouse were used as negative controls. Gates were established based on the distribution of the control cell groups and the numbers of cells in the experimental mice that fall into each group were determined.

## Results

Results of a recent study suggest that neointimal cells that form following vascular injury are derived from a stem cell population resident within the vascular wall rather than from previously differentiated VSMCs [6]. In that study fate mapping experiments were performed in which differentiated VSMCs were tagged using a cre-dependent EGFP reporter strain crossed with transgenic mice expressing cre recombinase exclusively in smooth muscle cells (B6.Cg-Tg(Myh11-cre,-EGFP)2Mik/J) [12]. The conclusion that previously differentiated VSMCs do not contribute to neointima formation was thus, based largely on the inability to detect EGFP-positive cells in the neointima in these mice. As there are many factors that can contribute to a negative result in these experiments, including the reported silencing of the ROSA 26 promoter that was used to drive EGFP expression in neointimal cells [13], we reevaluated these findings using an alternative fate mapping strategy. We utilized a dual color reporter transgenic mouse line (mTmG: B6.129(Cg)-Gt(ROSA)26Sor^tm4(ACTB-tdTomato,-EGFP)Luo^/J) in which all cells express a membrane localized mTomato tandem dimer in the absence of cre recombinase activity. Following cre-mediated excision of the mTomato cassette, cells express membrane localized EGFP [14] (Figure 1). In this reporter mouse line, both mTomato and mEGFP are driven by the same CMV/β-actin (CAG) promoter that has been shown to be active in neointimal cells [13]. This dual color reporter strain obviates the need to interpret negative data, as all neointimal cells should be either red or green depending on whether they express mTomato of mEGFP, respectively. To specifically identify differentiated VSMCs we crossed the mTmG mice with mice expressing a tamoxifen regulated cre recombinase directed by the smooth muscle-specific *Myh11*[8] or *Acta2*[9] promoters. The ability to temporally control cre recombinase activity also permitted us to distinguish previously differentiated VSMCs from newly differentiated VSMCs. Mice were treated with tamoxifen to activate the cre recombinase 2 weeks prior to vascular injury. As tamoxifen is rapidly metabolized in mice, at the time of surgical injury and subsequently, any cre recombinase expressed in VSMCs will be inactive and thus unable to switch cells from mTomato positive to mEGFP positive. Thus only VSMCs that were differentiated during the period of tamoxifen treatment, prior to injury will be mEGFP positive. As cre-mediated recombination results in a permanent heritable change in a cell’s genome these cells and any of their progeny will remain mEGFP positive even if they dedifferentiate and loose expression of cre or *Myh11 or Acta2*.

**Figure 2:**
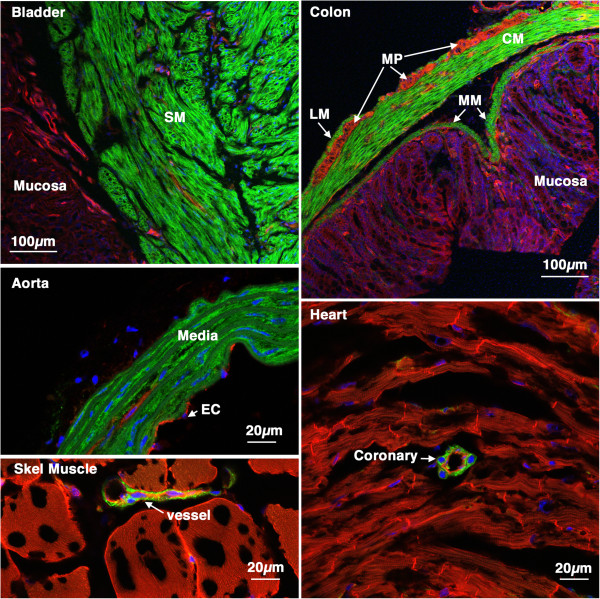
**Tissue specificity of *Myh11 *cre ER(T2) activity.** 5-week old *Myh11* creER(T2)^-/+^ mTmG^-/+^ mice were treated with tamoxifen (1 mg,IP) once a day for 5 days. 6 weeks later tissues were harvested and analyzed by confocal microscopy as described in ‘Methods’. A strong mEGFP signal can be seen only in smooth muscle cells of all the tissues examined including, bladder, colon, aorta, skeletal muscle and heart. No cre activity was detected in other cell types including skeletal muscle cells cardiac myocytes, endothelial cells or mucosal epithelial cells. SM-smooth muscle, LM-longitudinal muscle, CM-circular muscle, MP-myenteric plexus, MM-muscularis mucosa, EC-endothelial cells.

**Figure 3:**
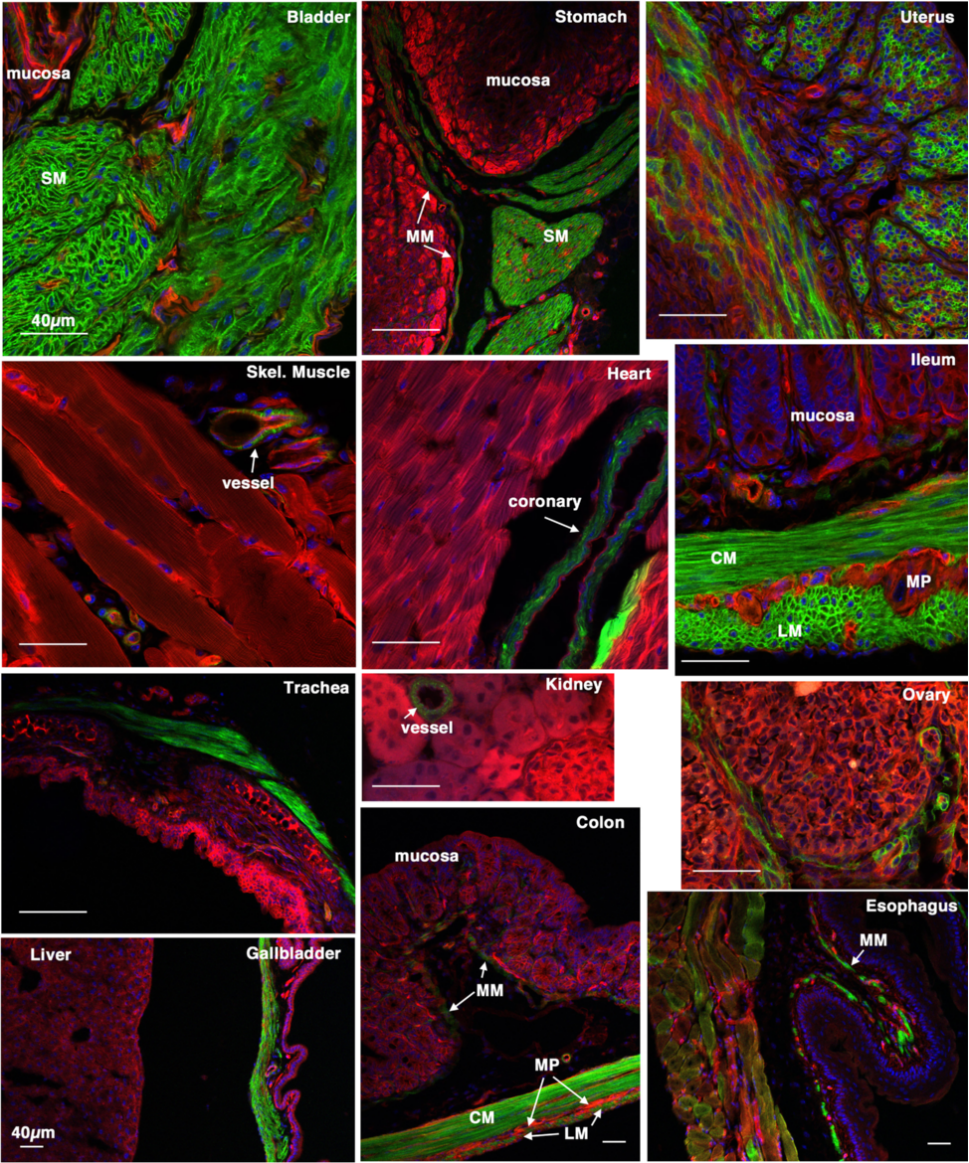
**Tissue specificity of *Acta2 *cre ER(T2) activity.** 5-week old *Acta2* creER(T2)^-/+^ mTmG^-/+^ mice were treated with tamoxifen (1 mg,IP) once a day for 5 days. 2 weeks following the last tamoxifen injection the left carotid artery was ligated. 4 weeks later tissues were harvested and analyzed by confocal microscopy as described in ‘Methods’. A strong mEGFP signal can be seen only in smooth muscle cells of all the tissues examined. No cre activity was detected in other cell types including skeletal muscle cells, cardiac myocytes, endothelial cells, mucosal epithelial cells and hepatocytes. A heterogeneous staining was observed in the uterus suggesting that cre was not active in all the uterine smooth muscle cells during the period in which the mice were treated with tamoxifen. The patchwork mEGFP expression observed in the esophagus reflects the mixed skeletal/smooth muscle lineage of cells within the wall of this portion of the esophagus. SM-smooth muscle, LM-longitudinal muscle, CM-circular muscle, MP-myenteric plexus, MM-muscularis mucosa. Scale bars represent 40 μm in all panels.

**Figure 4:**
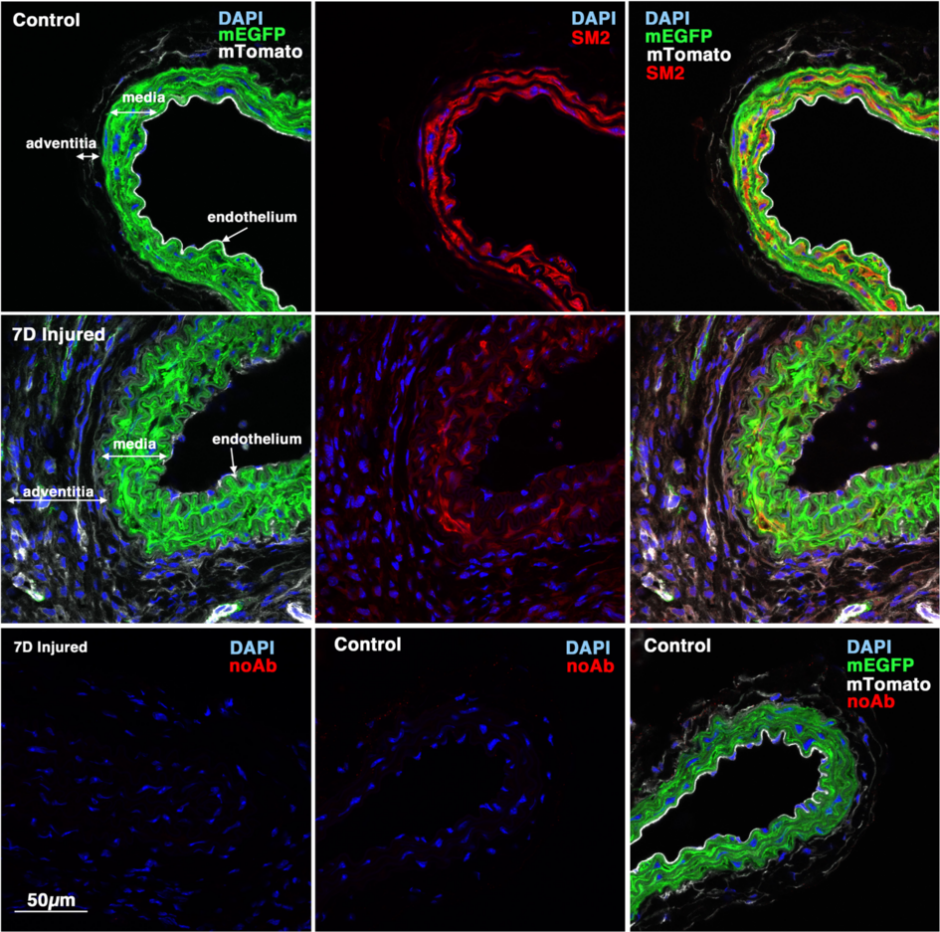
**Myh11 expression is down-regulated 7 days following injury.** 5-week old male *Myh11* creER(T2)^-/+^ mTmG^-/+^ mice were treated with tamoxifen (1 mg,IP) once a day for 5 days. Two weeks following the last tamoxifen injection the left carotid artery was ligated and tissues were harvested 7 days later. 8 μm cryosections obtained from injured and contralateral control arteries were analyzed for expression of *myh11* (using an anti-SM2 antibody) (red) and mEGFP (green) and mTomato (white). Control and injured sections are shown at identical exposures. Images shown are representative of those obtained from 3 different mice. In the lower panels the SM2 antibody was omitted and the samples otherwise processed identically to those shown in the upper panels. All images are shown at the same magnification with the scale bar shown in the bottom left panel representing 50 μm.

**Figure 5:**
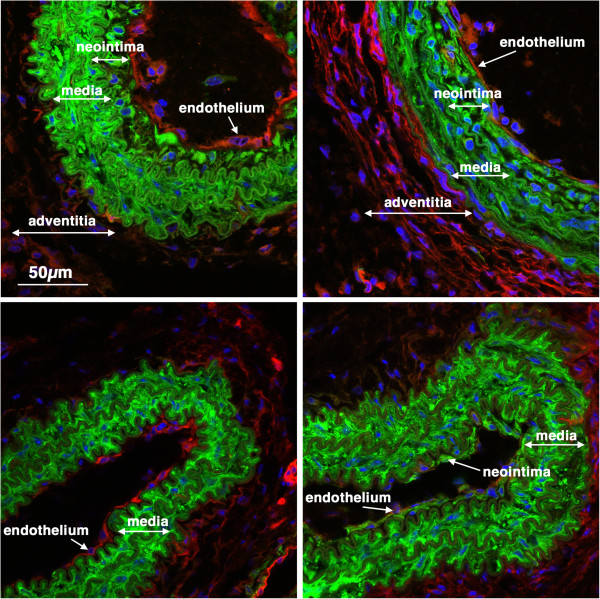
**Previously differentiated VSMC contribute to early neointima formation.** 5-week old male *Myh11* creER(T2)^-/+^ mTmG^-/+^ mice were treated with tamoxifen (1 mg,IP) once a day for 5 days. Two weeks following the last tamoxifen injection the left carotid artery was ligated and tissues were harvested 14 days later. Expression of mTomato (red) and mEGFP (green) were visualized in sections obtained from 4 different mice. Nuclei were visualized by staining with DAPI (blue). All images are shown at the same magnification with the scale bar shown in the top left panel representing 50 μm.

**Figure 6:**
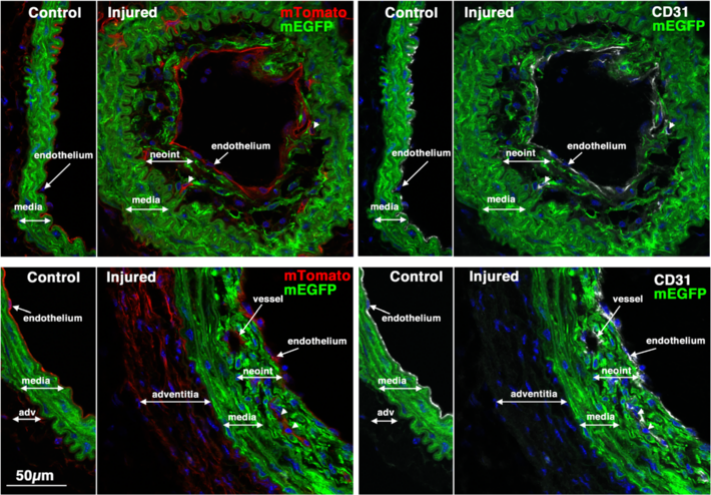
**CD31 positive endothelial cells can be seen within early developing neointima.** Sections from two of the mice shown in Figure 5 were stained with antibodies to CD31 (white). Left panels show mTomato (red)/mEGFP (green) co-stained sections and in the right panels are the same sections visualized for mEGFP (green) and CD31(white). Nuclei were visualized by staining with DAPI (blue). All images are shown at the same magnification with the scale bar shown in the bottom left panel representing 50 μm.

**Figure 7:**
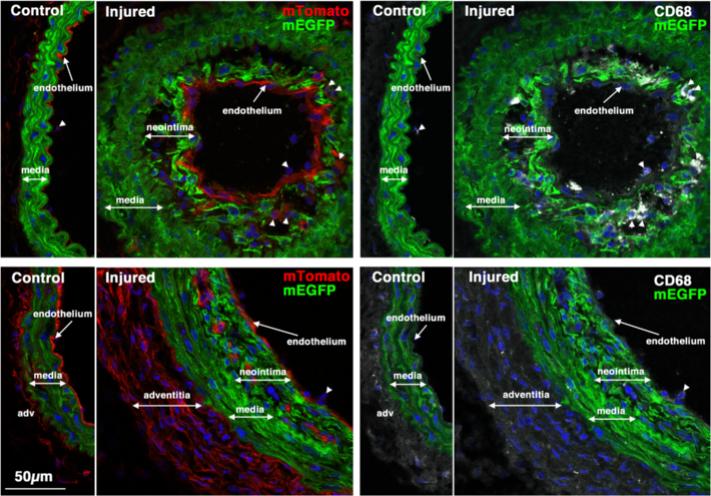
**CD68 positive macrophages/monocytes cells can be seen within early developing neointima in some mice.** Sections from the same two mice shown in Figure 6 were stained with antibodies to CD68 (white). Left panels show mTomato (red)/mEGFP (green) co-stained sections and in the right panels are the same sections visualized for mEGFP (green) and CD68(white). CD68-positive cells can be seen within the neointima of vessels from the mouse shown in the upper panels but not in the vessels of the mouse shown in the lower panels. Nuclei were visualized by staining with DAPI (blue). All images are shown at the same magnification with the scale bar shown in the bottom left panel representing 50 μm.

In *Myh11* creER(T2)^-/+^ mTmG^-/+^ double transgenic mice [8] and *Acta2* creER(T2)^-/+^ mTmG^-/+^[9] double transgenic mice (Figure 1) treatment with tamoxifen results in cre mediated recombination and thus subsequent expression of mEGFP specifically in vascular and visceral smooth muscle cells (Figures 2 and 3). No mEGFP expression was observed in endothelial cells, adventitial cells, cardiac or skeletal muscle cells (Figures 2 and 3). 7 days following injury in *Myh11* creER(T2)^-/+^ mTmG^-/+^ double transgenic mice we observed a significant decrease in expression of the endogenous *Myh11* gene within the medial layer of carotid arteries as evidenced by decreased smooth muscle myosin isoform, SM2, immunostaining (Figure 4). This is consistent with the previously reported dedifferentiation of medial VSMCs that follows vascular injury [15]. Despite the observed decreased *Myh11* expression, the medial mEGFP expression was similar in control and injured vessels 7 days following injury, suggesting that the CAG promoter, which drives mEGFP expression, is not affected by injury (Figure 4). At this time point we did not observe any significant neointima formation in any of the three mice examined. In contrast, 14 days following ligation we observed small amounts of neointima formation in 3 out of 4 mice examined (Figure 5). In these mice, mEGFP positive cells can be readily seen within the neointima suggesting that these cells are derived from previously differentiated (*Myh11* positive) medial VSMCs. Immunostaining with antibodies to CD31 demonstrate that there are also a number of CD31 positive, mTomato positive endothelial like cells within the neointima (Figure 6). We also observed some CD68 positive macrophage/monocytes in the neointima of some mice although these appeared less abundant than the CD31 positive cells (Figure 7).

**Figure 8:**
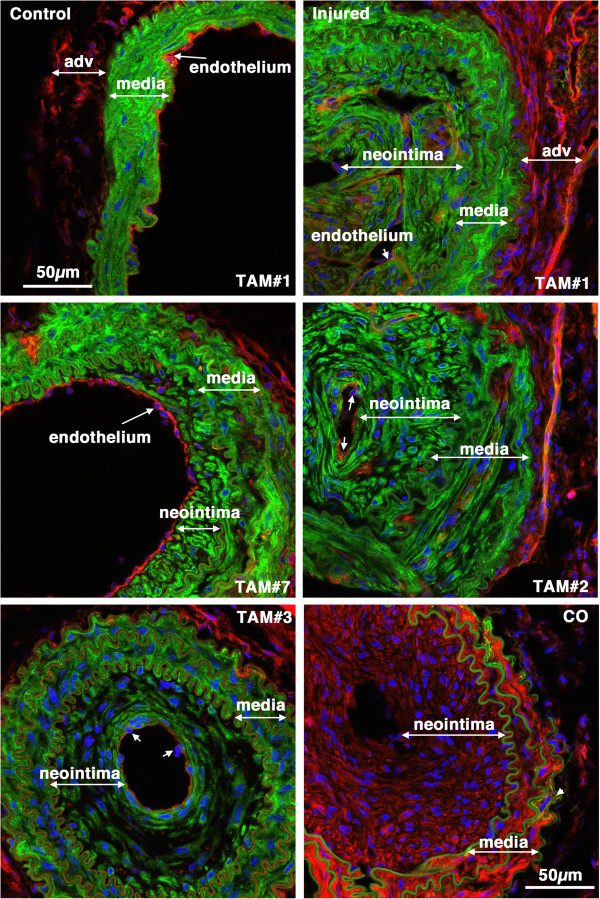
***Myh11*-tagged medial SMCs give rise to the mature neointima following carotid ligation.** Tissues were harvested 28 days following carotid ligation of 5-week old male *Myh11* creER(T2)^-/+^ mTmG^-/+^ mice that were previously Treated with Tamoxifen (TAM) or corn oil(CO) as indicated. In the upper two panels the control and injured carotid arteries from the same mouse are shown. The neointima, media and adventitia (adv) are indicated. Images are representative of those obtained from 7 tamoxifen treated mice (Mice numbers 1,7,2,3 in Table 1, as indicated). Arrows point to examples of endothelial cell nuclei. The image in the lower right hand panel is of an injured vessel obtained from a corn oil control treated mouse. The arrow-head in this image points to an mEGFP positive cell within the medial layer. All images are shown at the same magnification with the scale bars representing 50 μm.

**Figure 9:**
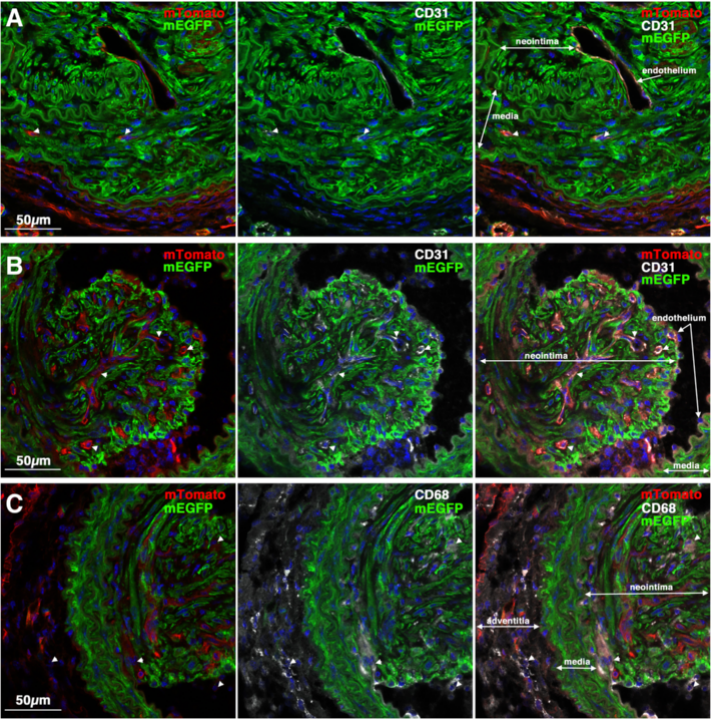
**CD31 and CD68 positive cells in mature neointimal lesions.** Images shown are examples of mature lesions seen in 2 different *Myh11* creER(T2)^-/+^ mTmG^-/+^ mice 28 days following ligation (Mouse 4(A) and 6(B, C); Table 1). **A**, **B**. Sections were immuno-stained for the endothelial/platelet marker CD31. Images shown are merged images showing the mTomato (red), mEGFP (green) and DAPI (blue); CD31 (white), mEGFP (green) and DAPI (blue) or mTomato, mEGFP, CD31 and DAPI channels, as indicated. Sections obtained from the mouse shown in panel A exhibited very few CD31 positive cells in the mature neointima; in contrast the lesion shown in panel B exhibited numerous CD31 positive, mTomato positive, mEGFP negative cells in the neointima (examples indicated by arrow heads). **C**. Sections (serial to those shown in panel B) were immuno-stained for the macrophage/monocyte marker CD68. Images shown are merged images showing the mTomato (red), mEGFP (green) and DAPI (blue); CD68 (white), mEGFP (green) and DAPI (blue) or mTomato, mEGFP, CD68 and DAPI channels, as indicated. Several CD68 positive, mTomato positive, mEGFP negative cells can be seen in the neointima and adventitia (examples indicated by arrow heads). No CD68 positive cells were seen in the neointima of the mouse shown in panel A (data not shown).

To better quantitate the contribution of mEGFP positive cells to neointima formation we examined more mature lesions that formed 28 days following ligation. In most *Myh11* creER(T2)^-/+^ mTmG^-/+^ double transgenic mice 28 days following carotid ligation the majority of neointimal cells also expressed mEGFP (Figure 8, Table 1). However, in one mouse the neointima was comprised of only 42% of mEGFP positive cells (Mouse#6 in Table 1, Figure 9B, C). In this mouse the lesion was very unusual in that it extended out in a bulb like structure from one side of the vessel wall (Figure 9B). Many of the mTomato positive cells in this lesion also stained positive for either CD31 or CD68 suggesting a large number of endothelial cells and macrophages or monocytes in the lesion (Figure 9B, C). In contrast, no CD68 positive cells were detected in most mature lesions and only a few scattered endothelial cells were seen within these lesions, although they could be readily seen lining the residual vessel lumens (Figure 9A). In another mouse, not shown in Table 1, we did not observe any concentric neointima formation 28 days following injury. In contrast, in this mouse there was an mEGFP negative cluster of CD31 positive cells that covered approximately 50% of the vessel lumen. As this structure occurred close to the point of ligation (<100 μm away), was very focal only extending for about 300 μm, was atypical in shape and comprised of CD31 positive cells we speculate that it was an organized thrombus rather than a true neointimal lesion. This mouse was thus excluded from our analyses. In control mice, given corn oil instead of tamoxifen, almost all cells remained mTomato positive and mEGFP negative (Figure 8, lower right panel). One mEGFP positive cell can be seen in the media of the corn oil treated vessel (green/yellow cell indicated by arrow head). This cell likely represents one in which the creER(T2) protein has moved to the nucleus in the absence of the tamoxifen ligand. As this cell also seems to express some mTomato (accounting for its yellow color) it is likely that the recombination event occurred within a few days of tissue harvesting such that the residual mTomato protein has not had time to completely turn over and disappear from the cell.

**Table 1:** Quantitation of mEGFP positive neointimal cells 28 days following ligation

***Myh11 *** **cre**	**% mEGFP +**	**Averaged from:**
Mouse #		
1	76 ± 9	1827 cells (18 stacks, 10 sections)
2	86 ± 4	600 cells (5 stacks, 3 sections)
3	80 ± 11	773 cells (12 stacks, 8 sections)
4	90 ± 5	653 cells (6 stacks, 4 sections)
5	92 ± 4	227 cells (5 stacks, 3 sections)
6	42 ± 10	517 cells (5 stacks, 3 sections)
7	85 ± 7	463 cells (10 stacks, 8 sections)
Mean	79 ± 17	5060 cells (61 stacks, 39 sections)
***Acta2 *** **cre**	**% mEGFP+**	**Averaged from:**
Mouse#		
8	91 ± 4	731 cells (5 stacks, 3 sections)
9	72 ± 13	208 cells (8 stacks, 6 sections)
10	63 ± 16	189 cells (10 stacks, 6 sections)
11	74 ± 4	261 cells (6 stacks, 3 sections)
12	81 ± 7	561 cells (5 stacks, 4 sections)
13	94 ± 7	759 cells (8 stacks, 8 sections)
14	92 ± 3	190 cells (4 stacks, 4 sections)
Mean	81 ± 12	2899 cells (46 stacks, 34 sections)

In order to count the number of mEGFP and mTomato positive cells within the neointima of each vessel (the cells between the internal elastic lamina and the endothelial lining of the lumen), a Z-stack series of 1 μm optical sections (XY-210 μm × 210 μm) were obtained from each vessel. mEGFP and mTomato positive cells were counted in each of the optical sections through the entire Z-stack of images, each DAPI positive nuclei was scored as being associated with either an mEGFP or mTomato positive cell. The number of total cells counted, together with the number of separate Z-stacks and sections counted are indicated. From some sections we were able to obtain 2 separate fields of Z-stack images, in others a single field encompassed the entire neointima.

**Figure 10:**
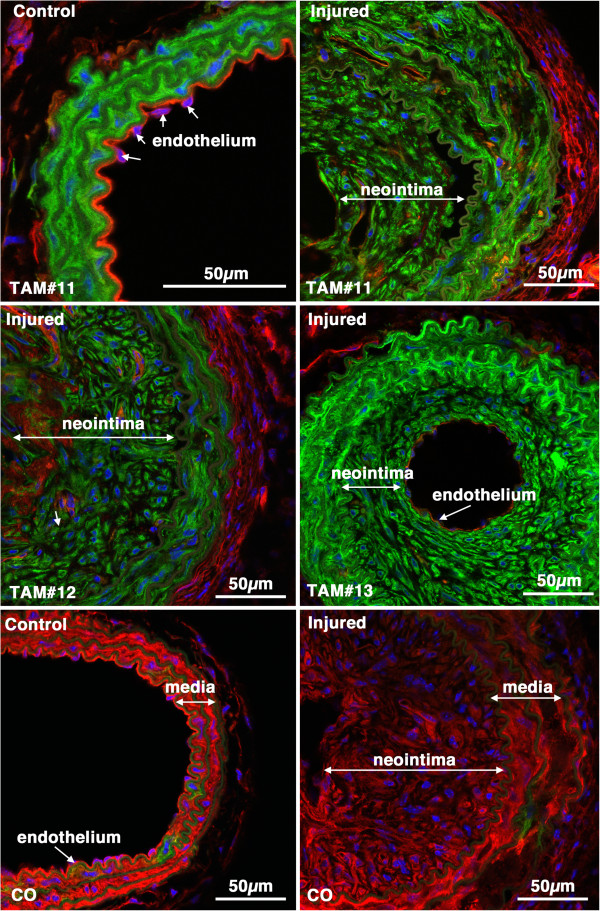
***Acta2*-tagged medial SMCs give rise to the neointima following carotid ligation.** Control and injured carotid arteries from Tamoxifen (TAM) or corn oil (CO) treated *Acta2* creER(T2)^-/+^ mTmG^-/+^ transgenic mice harvested 28 days post ligation (Mice numbers 11, 12 and 13, respectively; Table 1). Images from tamoxifen treated injured vessels are representative of those obtained from 7 mice.

**Figure 11:**
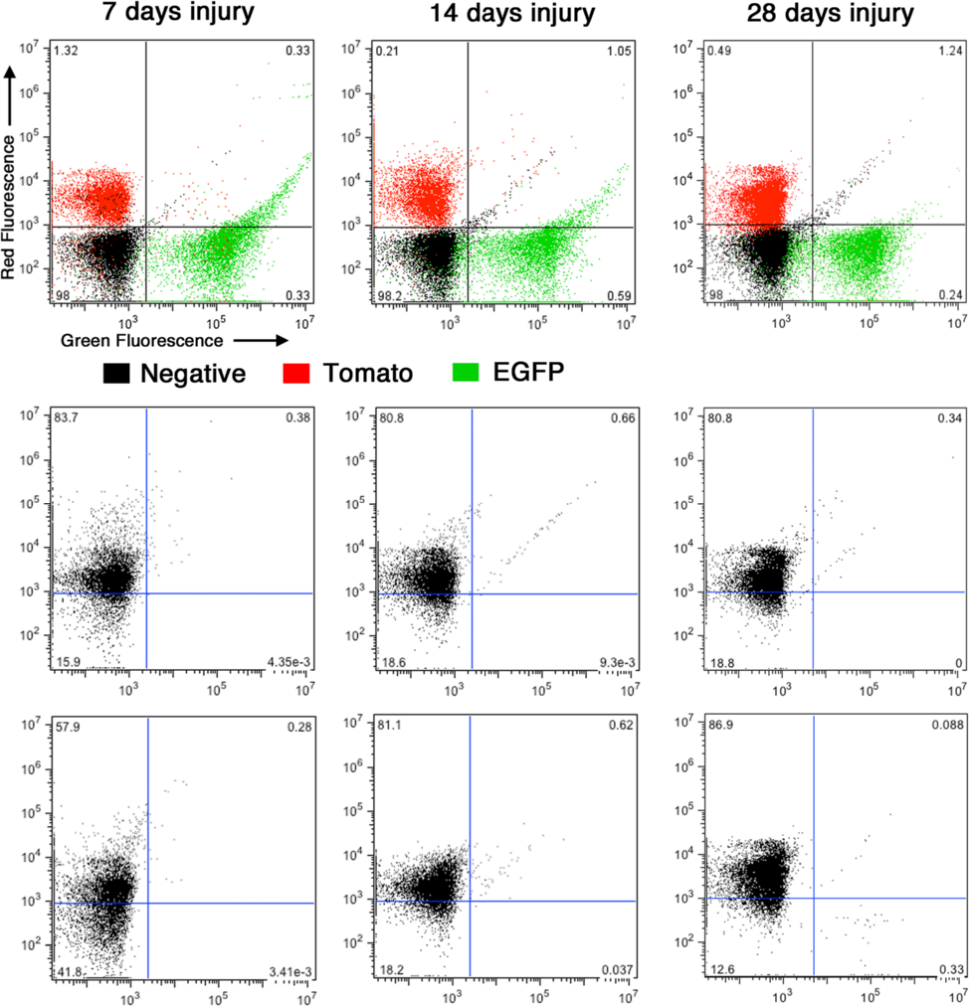
**No mEGFP positive circulating cells can be detected following carotid ligation.** Blood was harvested from *Myh11* creER(T2)^-/+^ mTmG^-/+^ mice 7, 14 and 28 days following carotid ligation and subjected to fluorescence activated cell sorting. Cells harvested from a CAG-EGFP mouse were used as positive controls for EGFP expressing cells, those harvested from mTmG reporter mice with no cre transgene as positive controls for mTomato positive cells and those harvested from nontransgenic mice as negative controls. From each mouse a minimum of 10,000 cells were sorted. Upper panels show the distribution of mTomato (red) and mEGFP (green) positive as well as negative (black) cells. Numbers on each graph show the percentage of negative cells in each quadrant. Lower panels show the distribution of cells obtained from duplicate *Myh11* creER(T2)^-/+^ mTmG^-/+^ mice. In each case the percentage of EGFP-positive cells are less than background negative control levels.

Consistent with data obtained from the *Myh11* cre mice, when previously differentiated VSMCs were tagged with the use of *Acta2* cre the majority of the neointimal cells were mEGFP positive (range 63-94%; mean of 81 ± 12%; Figure 10, Table 1). Although the data presented suggest that many neointimal cells are derived from previously differentiated (*Myh11* or *Acta2* positive) medial VSMCs it is possible that these cells may be mobilized from distant sites rather than being derived from locally resident medial VSMCs. To evaluate the existence of a population of mobile mEGFP positive cells we performed a fluorescence activated cell sorting (FACS) analysis of blood obtained from tamoxifen treated *Myh11* creER(T2)^-/+^ mTmG^-/+^ double transgenic mice, 7,14 and 28 days following injury (Figure 11). This analysis failed to detect the presence of any significant numbers of mEGFP positive circulating cells suggesting that the mEGFP positive neointimal cells are most likely locally derived.

## Discussion

Results from the current studies support the widely accepted paradigm that following vascular injury medial VSMCs dedifferentiate and migrate into the lumen of vessels forming a neointima. As we utilized a tamoxifen regulated cre recombinase and waited 2 weeks after tamoxifen treatment before performing carotid ligation, given the 12 hour half life of tamoxifen in serum, it is highly unlikely that mEGFP positive cells seen following injury are derived from newly differentiated cells. Moreover, the use of a dual color reporter system avoided any artifacts that may arise due to promoter or reporter silencing as all cells should be either mTomato or mEGFP positive thus no conclusions need to be drawn that are based on negative staining data. Our data also suggest that unlike the ROSA-LACz reporter gene [13] the CMV enhancer/chicken beta-actin core promoter (CAG) driven mTmG reporter gene is not down-regulated in neointimal SMCs (compare the mEGFP intensity of control and injured vessels in Figures 3, 8 and 10). The use of the mTmG reporter strain has the additional advantage that the reporter proteins are membrane localized and thus better retained during sample processing. Moreover, this is a single copy, targeted transgene, hence, it is also not subject to complications that may arise from partial recombination of multicopy transgenes, such that in each cell’s nucleus either the mTomato gene is present or it is excised. Cells will thus express either mTomato or mEGFP. Anecdotally we have noted, that the mTomato protein is relatively stable such that after tamoxifen treatment smooth muscle cells can have detectable expression of both mTomato and mEGFP for 3-4 days before the mTomato protein is turned over and degraded. We speculate that one or more of these advantages of the mTmG reporter system and tamoxifen regulated cre transgenes used in our study may explain why we were able to detect mEGFP positive neointimal cells whereas they were not detected in a previous study [6].

Our data are consistent with and extend previous fate mapping studies using a cre-dependent LacZ reporter [5]. In this study a cre-dependent ROSA-LacZ reporter was used together with *Myh11*-creER(T2) transgenic mice to show that following femoral artery wire injury the neointima that formed contained LacZ positive cells [5]. Together these studies suggest that in both the small concentric lesions that form following femoral wire injury and the more complex large lesions that form following carotid ligation, neointimal cells arise from differentiated medial VSMCs. Our rigorous quantitative analysis using 1 μm optical sections to obtain Z-stack series through the entire thickness of each 8 μm section, revealed some heterogeneity between mice, with mEGFP positive cells comprising 42-94% of total neointimal cells (Table 1). Although most mice have between 70-90% mEGFP positive neointimal cells, this may perhaps account for some of confusion in the literature related to the contribution of different cell types to the neointima. Despite this variability, the data indicate that, on average, the majority (~80%) of neointimal cells arise from the previously differentiated medial SMCs. This number would be consistent with previous studies that showed that about 20% of neointimal cells are derived from blood borne cells [4]. In further support of the contribution of blood or endothelial derived cells to the neointima some of the mTomato positive, mEGFP negative neointimal cells observed in our study, stained positive for endothelial and monocyte markers (CD31 and CD68, respectively; Figures 6, 7 and 9). It is perhaps a little surprising that in some lesions there were more CD31 positive endothelial cells within the lesions than CD68 positive monocytes/macrophages. Some of these CD31 positive endothelial cells may be present in new vessels that are growing within the neointima (e.g. Figure 6, lower panels) and some may be miscounted lumen endothelial cells in which the plane of the section has obscured a luminal invagination. We also speculate that the proliferating neointimal smooth muscle cells may trap endothelial cells within the lesion as they extend out into the vessel lumen.

## Conclusions

Although our studies do not rule out the possibility that under appropriate, in vitro, culture conditions the expansion of a progenitor cell population may be favored, the current studies provide compelling evidence that, in vivo, the majority of neointimal cells that arise following carotid ligation are derived from differentiated medial VSMCs. The lack of detectable mEGFP positive cells circulating in the blood, further suggests that the neointimal cells likely arise from the dedifferentiation and migration of locally derived medial VSMCs.

## Competing interests

The authors declare that they have no competing interests.

## Authors’ contributions

BPH prepared the manuscript and figures, harvested tissues, obtained cryosections, performed all confocal microscopy, immunohistology and quantitative analysis. AMH maintained mouse lines, performed carotid ligations and harvested tissues. CB provided access and training in the use of the confocal microscope and performed FACS studies. SO generated the *myh11* creER(T2) transgenic mice, aided in data interpretation and reviewed and edited the final manuscript. All authors reviewed and approved the final manuscript.
